# Gartner’s Duct Cyst Arising From the Posterolateral Rectovaginal Pouch: An Unusual Location

**DOI:** 10.7759/cureus.105737

**Published:** 2026-03-23

**Authors:** Harshil Anand, Prashant Onkar, Suresh Phatak, Kajal Mitra, Pranit B Pantawane

**Affiliations:** 1 Department of Radiodiagnosis, N. K. P. Salve Institute of Medical Sciences and Research Centre and Lata Mangeshkar Hospital, Nagpur, IND

**Keywords:** benign cystic lesions, cervicovaginal junction, cystic lesion of cervix, douglas pouch, gartner's duct cyst, pelvic discomfort, persistent white vaginal discharge, posterior lip of cervix, rectovaginal pouch, unusual location

## Abstract

Gartner’s duct cyst is a benign cystic lesion arising from persistent remnants of the mesonephric (Wolffian) duct in females. It is most commonly located along the anterolateral vaginal wall and is often detected incidentally. Presentation in the posterolateral aspect of the rectovaginal pouch is uncommon. We report the case of a 32-year-old multiparous woman who presented with chronic vaginal discharge and pelvic discomfort and was found to have a Gartner’s duct cyst located in the left posterolateral rectovaginal pouch, extending superiorly to the posterior lip of the cervix. Radiological evaluation with ultrasound and MRI suggested a benign cystic lesion, and histopathological examination confirmed the diagnosis. This case highlights an unusual anatomical location of a Gartner’s duct cyst and underscores the importance of considering it in the differential diagnosis of posterior vaginal and rectovaginal cystic lesions.

## Introduction

Gartner’s duct cysts represent benign cystic formations derived from residual mesonephric (Wolffian) duct tissue that fails to regress completely during female embryologic development [1-4]. Although these remnants are not uncommon, cystic transformation is relatively infrequent and most often involves the anterolateral vaginal wall [1-4]. Posterior or rectovaginal involvement is uncommon and less frequently described in the literature [5]. In many cases, such lesions remain small and asymptomatic and are identified incidentally during routine gynecologic evaluation [1-4].

Although most Gartner’s duct cysts remain clinically silent, larger lesions may present with vaginal discharge, dyspareunia, pelvic pain, urinary complaints, or a sensation of vaginal fullness [1-3,6]. Due to their uncommon location and nonspecific presentation, posteriorly located Gartner’s duct cysts may pose a diagnostic challenge because of their proximity to adjacent pelvic structures and overlap with other cystic pathologies. Recognition of this atypical presentation is therefore important for accurate diagnosis and appropriate management. We report a case of a Gartner’s duct cyst arising from the left posterolateral rectovaginal pouch, extending up to the posterior lip of the cervix.

## Case presentation

A 32-year-old woman, gravida 2, para 1, with one living child and one prior abortion, presented to the outpatient gynecology clinic with a history of persistent white vaginal discharge for one to two years, associated with lower backache and occasional dysuria. There was no history of fever, weight loss, or bowel complaints. She had undergone surgery eight years earlier for an ovarian cyst. Her menstrual cycles were regular, occurring every 30-35 days, lasting five to six days, with moderate flow and associated dysmenorrhea.

Clinical examination, including general and systemic assessment, revealed no abnormalities. On speculum examination, a cystic swelling was noted on the posterior aspect of the cervix, extending toward the pouch of Douglas. Per vaginal examination revealed a parous cervical os deviated to the left, and a cystic lesion was palpated in the posterior fornix.

Routine laboratory investigations were within normal limits (Table 1).

**Table 1:** Routine laboratory investigations

Parameter	Patient value	Reference range
Hemoglobin	15.5 g/dL	12-16 g/dL
Total leukocyte count	6,180/mm³	4,000-11,000/mm³
Platelet count	1.81 × 10⁵/mm³	1.5-4.5 × 10⁵/mm³
Blood urea	14 mg/dL	6-21 mg/dL
Serum creatinine	0.4 mg/dL	0.6-1.1 mg/dL
Urine color	Yellow	Pale yellow to yellow
Urine pH	5.5	5.0-8.0
Specific gravity	1.010	1.005-1.030
Protein	Negative	Negative
Glucose	Negative	Negative
RBCs	0/HPF	0-3/HPF
WBCs (pus cells)	0/HPF	0-5/HPF

All urine routine examination parameters were within normal limits, with no evidence of hematuria, pyuria, proteinuria, or glycosuria.

**Figure 1:**
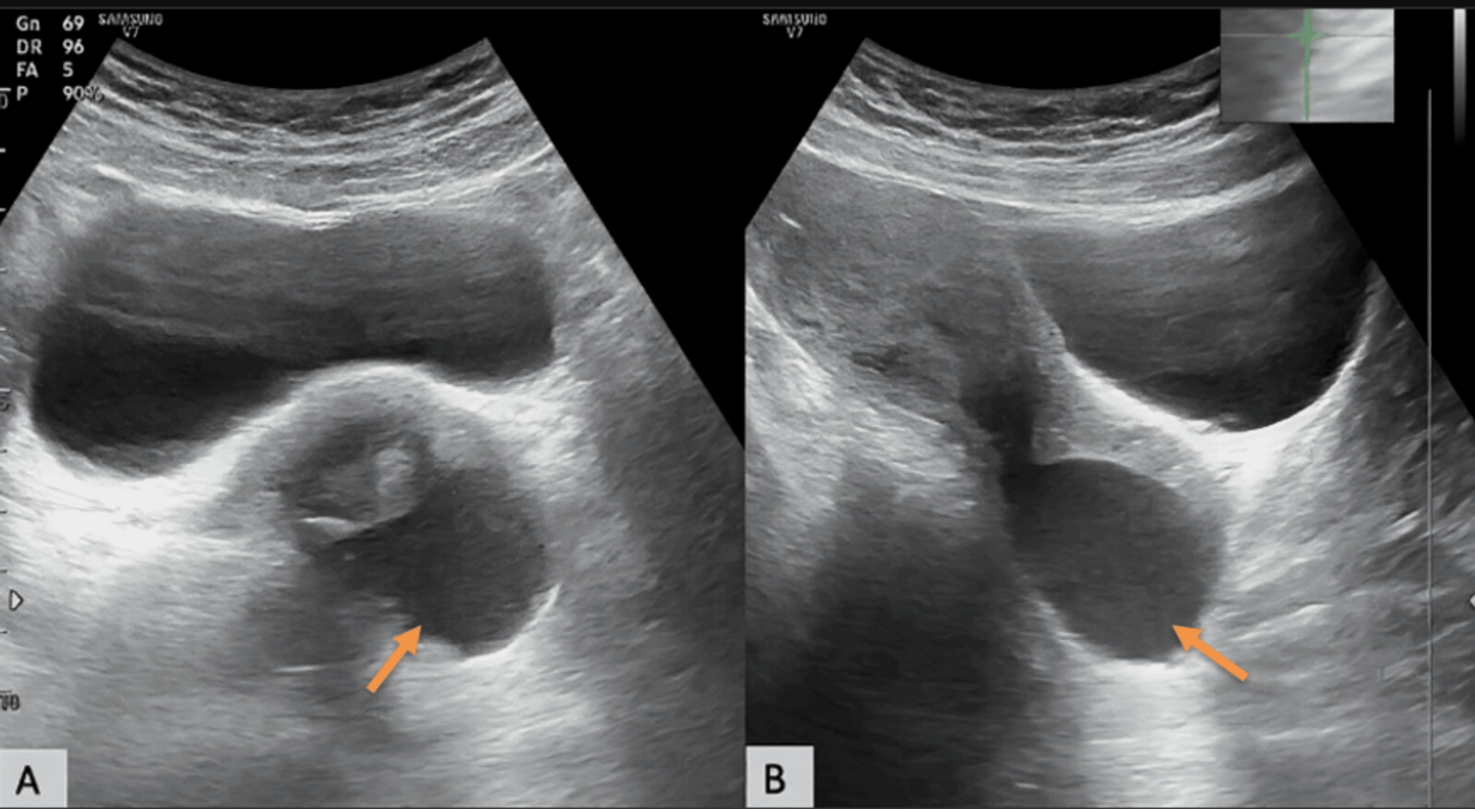
Transabdominal USG images (A) Axial and (B) sagittal gray-scale images demonstrating a well-defined anechoic cystic lesion (orange arrows) at the cervicovaginal junction involving the posterior lip of the cervix.

**Figure 2:**
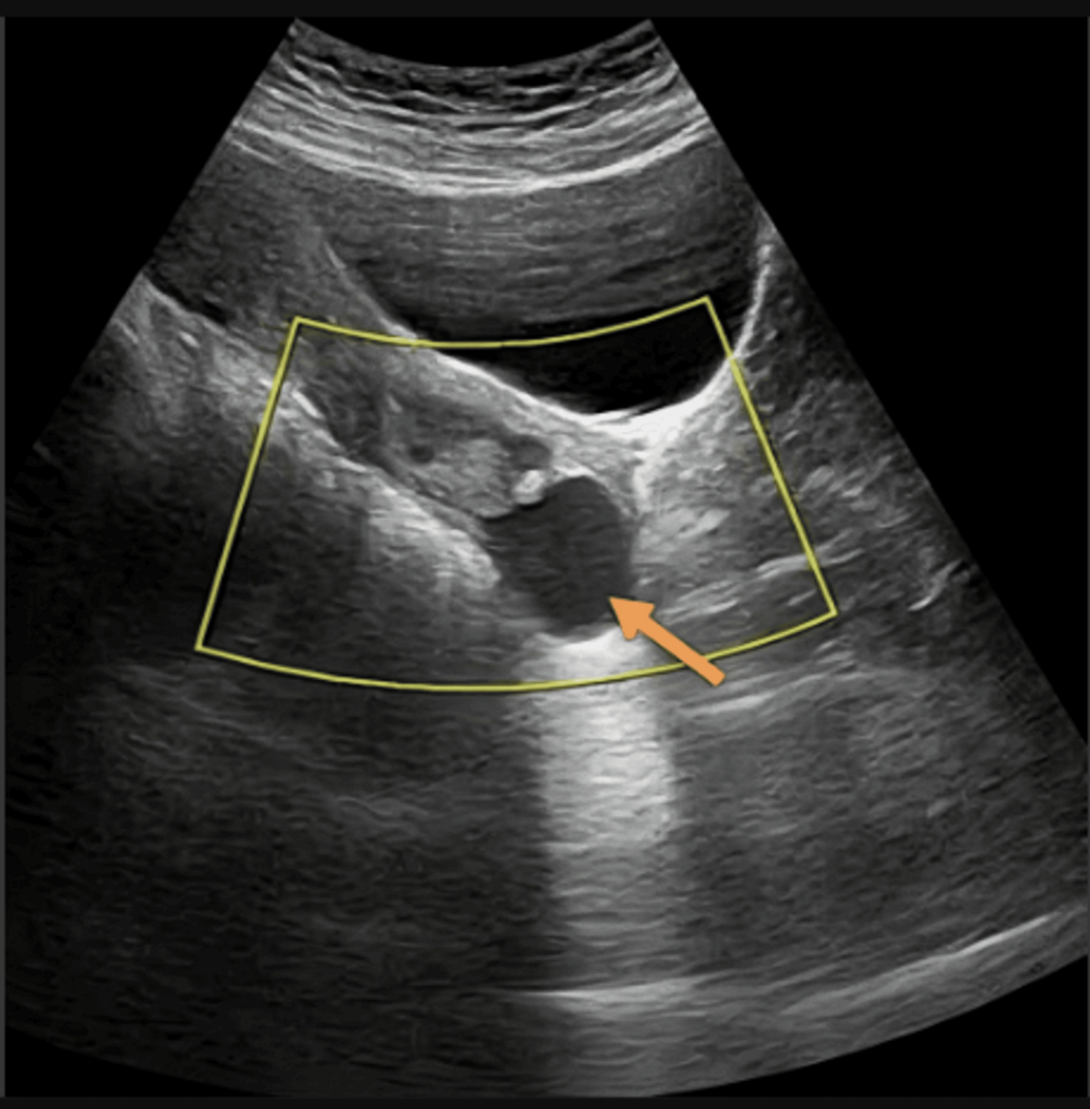
Sagittal color Doppler USG image Color box showing absence of internal vascularity within the lesion (orange arrow).

Transabdominal USG demonstrated a well-defined anechoic cystic lesion at the cervicovaginal junction involving the posterior lip of the cervix (Figure 1A, 1B). No internal vascularity was observed on color Doppler imaging (Figure 2).

**Figure 3:**
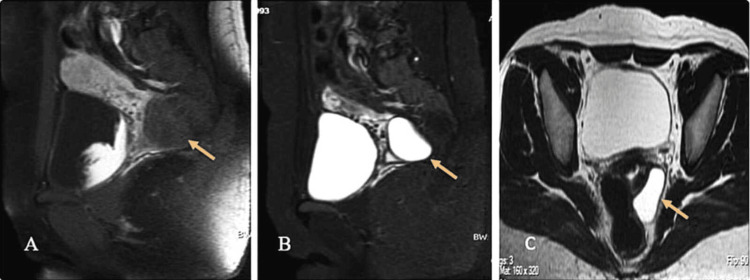
MRI of the pelvis (A) Sagittal T1-weighted and (B) sagittal STIR images demonstrating a well-defined cystic lesion (orange arrows) in the left posterolateral rectovaginal pouch, extending to the posterior lip of the cervix, appearing hypointense on T1-weighted and hyperintense on STIR images. (C) Axial T2-weighted image demonstrating a hyperintense cystic lesion in the left posterolateral rectovaginal pouch (orange arrow). STIR, short tau inversion recovery

MRI revealed a well-defined cystic lesion with insinuating margins in the left posterolateral aspect of the rectovaginal pouch, extending superiorly to the posterior lip of the cervix. The lesion appeared hypointense on T1-weighted imaging and hyperintense on T2-weighted and short tau inversion recovery sequences, without diffusion restriction or post-contrast enhancement (Figure 3A-3C).

**Figure 4:**
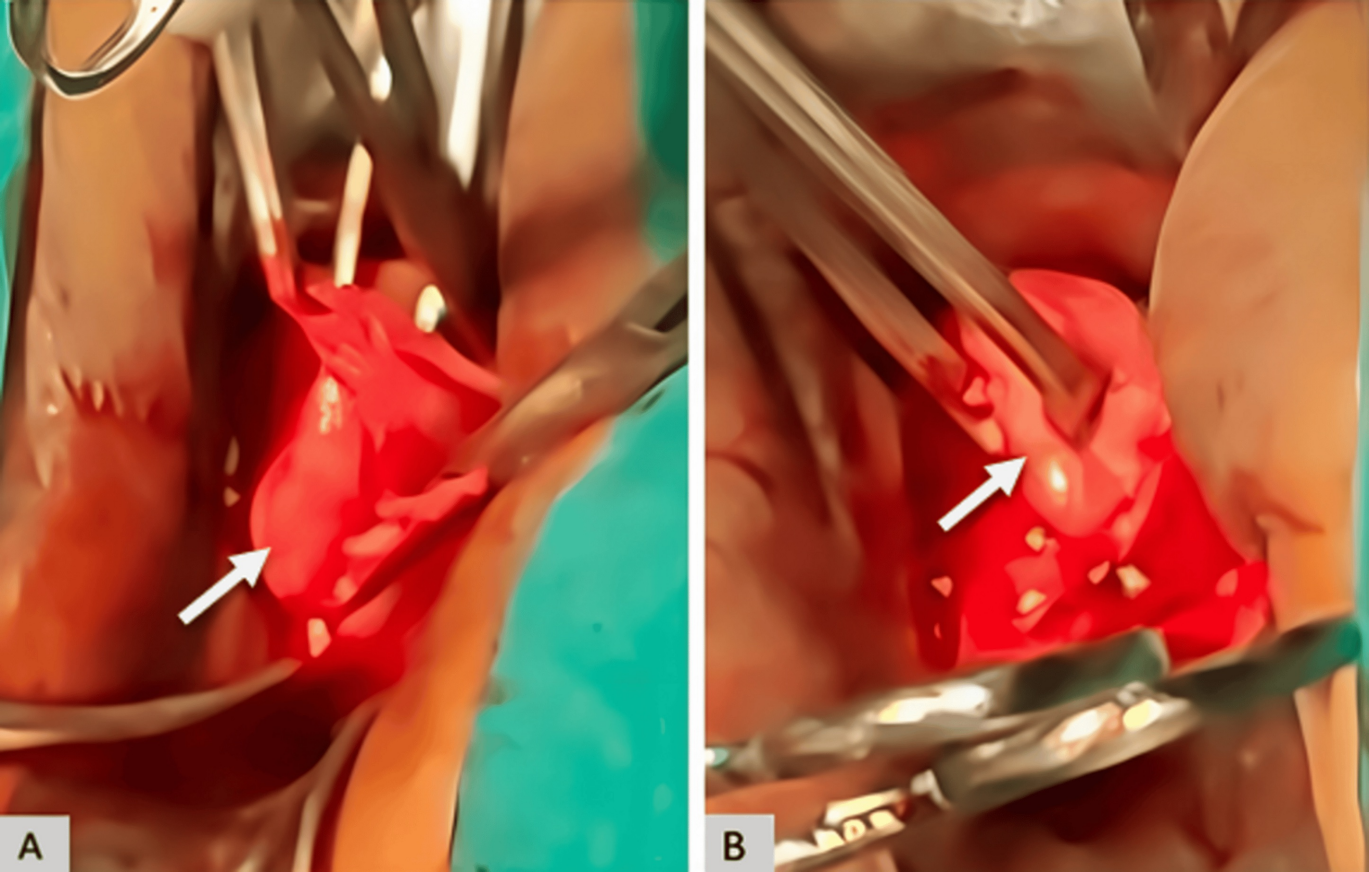
Intraoperative images (A) A well-defined, smooth cystic lesion (white arrow) visualized during surgical exposure, corresponding to the lesion identified on preoperative imaging. (B) The lesion (white arrow) being grasped with forceps prior to dissection.

The patient underwent elective surgical excision. Intraoperatively, a cystic lesion was identified (Figure 4A, 4B) arising from the pouch of Douglas and involving the posterior lip of the cervix. Dissection of the cyst wall was attempted; however, complete separation was not feasible. Tissue samples from the cyst wall and adjacent vaginal wall were submitted for histopathological examination.

**Figure 5:**
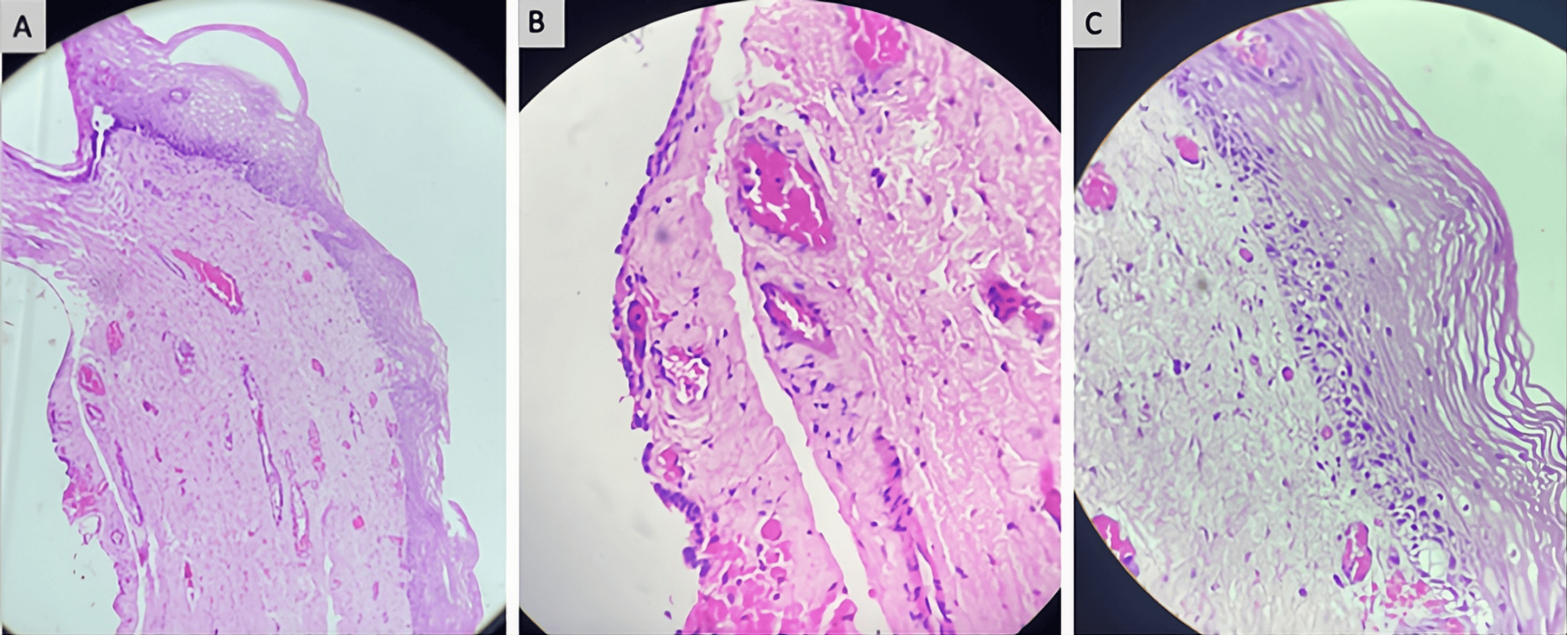
Histopathological examination of the excised cyst wall (H&E stain) (A) Low-power photomicrograph demonstrating cyst wall architecture with partial epithelial lining. (B) Higher magnification showing cuboidal epithelial lining overlying fibrovascular stroma with chronic inflammatory infiltrate. (C) Stratified squamous epithelium exhibiting acanthosis with focal basal nuclear atypia and areas of epithelial flattening.

Microscopic examination revealed a cyst wall partially lined by cuboidal epithelium. The overlying stratified squamous epithelium demonstrated acanthosis with anisonucleosis in the lower layers. Areas of epithelial flattening were also observed. The subepithelial tissue consisted of fibrovascular stroma with chronic inflammatory infiltrates. These findings were consistent with a Gartner’s duct cyst, with focal mild dysplasia noted in the overlying epithelium (Figure 5A-5C).

The postoperative course was uneventful, and the patient was discharged in stable condition.

## Discussion

Gartner’s ducts are identified in approximately 25% of adult women, although only 1-2% progress to form clinically detectable cysts [2,3]. During embryogenesis, the mesonephric ducts regress in females; incomplete regression may result in the formation of Gartner’s duct cysts. Classically, these cysts are small (average diameter of 2 cm), solitary, unilateral, and located along the anterolateral vaginal wall [1-4].

Posterior or rectovaginal locations are uncommon and may mimic other cystic lesions of the posterior vaginal compartment, including endometriotic cysts, Müllerian cysts, and inclusion cysts. Accurate radiologic characterization is therefore essential in narrowing the differential diagnosis.

On USG, a Gartner’s duct cyst typically appears as a well-circumscribed, thin-walled, anechoic lesion without internal septations, mural nodules, or internal vascularity on color Doppler imaging [7]. In contrast, endometriotic cysts often demonstrate internal low-level echoes due to hemorrhagic content, sometimes described as a “ground-glass” appearance [8]. Inclusion cysts may show variable echogenic debris depending on keratin content [9].

MRI provides superior soft-tissue contrast and precise anatomical localization. A Gartner’s duct cyst typically demonstrates low signal intensity on T1-weighted imaging and high signal intensity on T2-weighted imaging, consistent with simple fluid content [10]. The absence of diffusion restriction and post-contrast enhancement supports a benign cystic nature. In contrast, endometriotic cysts frequently show T1 hyperintensity due to blood products and may exhibit T2 shading from chronic hemorrhage [11]. Müllerian cysts may have variable signal characteristics depending on mucinous content but remain confined to the vaginal wall [4].

A definitive diagnosis is established by histopathologic examination, which typically reveals a cyst lined by cuboidal or columnar epithelium, with or without squamous metaplasia. Surgical excision is indicated in symptomatic cases or when malignancy cannot be excluded.

## Conclusions

This case highlights an uncommon presentation of a Gartner’s duct cyst arising from the posterolateral aspect of the rectovaginal pouch, extending up to the posterior lip of the cervix. Awareness of such atypical locations is essential to avoid misdiagnosis and to guide appropriate management. Imaging, particularly MRI, combined with histopathological confirmation, remains the cornerstone of diagnosis.

## References

[1] Bala R, Nagpal M, Kaur M, Kaur H (2015). Posterior vaginal wall Gartner's duct cyst. J Midlife Health.

[2] Letizia MJ, Kelly J (2011). Case report: Gartnerʼs duct cyst. Emerg Med News.

[3] Siegelman ES, Outwater EK, Banner MP, Ramchandani P, Anderson TL, Schnall MD (1997). High-resolution MR imaging of the vagina. Radiographics.

[4] Eilber KS, Raz S (2003). Benign cystic lesions of the vagina: a literature review. J Urol.

[5] Kondi-Pafiti A, Grapsa D, Papakonstantinou K, Kairi-Vassilatou E, Xasiakos D (2008). Vaginal cysts: a common pathologic entity revisited. Clin Exp Obstet Gynecol.

[6] Memon SI, Acharya N (2022). A rare case of posterior vaginal wall Gartner's duct cyst mimicking as genital prolapse. Cureus.

[7] Scheible FW (1978). Ultrasonic features of Gartner's duct cyst. J Clin Ultrasound.

[8] Van Holsbeke C, Van Calster B, Guerriero S (2010). Endometriomas: their ultrasound characteristics. Ultrasound Obstet Gynecol.

[9] Lee HS, Joo KB, Song HT (2001). Relationship between sonographic and pathologic findings in epidermal inclusion cysts. J Clin Ultrasound.

[10] Elsayes KM, Narra VR, Dillman JR, Velcheti V, Hameed O, Tongdee R, Menias CO (2007). Vaginal masses: magnetic resonance imaging features with pathologic correlation. Acta Radiol.

[11] Tanaka YO, Okada S, Yagi T, Satoh T, Oki A, Tsunoda H, Yoshikawa H (2010). MRI of endometriotic cysts in association with ovarian carcinoma. AJR Am J Roentgenol.

